# X-Ray Phase Nanotomography Resolves the 3D Human Bone Ultrastructure

**DOI:** 10.1371/journal.pone.0035691

**Published:** 2012-08-29

**Authors:** Max Langer, Alexandra Pacureanu, Heikki Suhonen, Quentin Grimal, Peter Cloetens, Françoise Peyrin

**Affiliations:** 1 Creatis, Université de Lyon, CNRS UMR5220, Inserm U1044, INSA-Lyon, Université Lyon 1, Lyon, France; 2 European Synchrotron Radiation Facility, Grenoble, France; 3 LAPI, Politehnica University of Bucharest, Bucharest, Romania; 4 LIP, UPMC Université Pierre et Marie Curie Paris 6, CNRS UMR7623, Paris, France; Institute of Psychology, Chinese Academy of Sciences, China

## Abstract

Bone strength and failure are increasingly thought to be due to ultrastructural properties, such as the morphology of the lacuno-canalicular network, the collagen fiber orientation and the mineralization on the nanoscale. However, these properties have not been studied in 3D so far. Here we report the investigation of the human bone ultrastructure with X-ray phase nanotomography, which now provides the required sensitivity, spatial resolution and field of view. The 3D organization of the lacuno-canalicular network is studied in detail over several cells in osteonal and interstitial tissue. Nanoscale density variations are revealed and show that the cement line separating these tissues is hypermineralized. Finally, we show that the collagen fibers are organized as a twisted plywood structure in 3D.

## Introduction

Bone is a hierarchically organized, multiscale natural nanocomposite that is stiff and tough while maintaining lightness. It is also a dynamical tissue that detects and repairs damage at all length scales, thereby adapting to external mechanical constraints [1], [2]. The mechanisms enabling and controlling these processes are not entirely understood, mainly due to the complex, multiscale 3D organization combined with mechanical and biochemical processes at the cellular level. Bone fragility disease is generally associated with a disturbance of the bone remodeling process, disrupting the balance between tissue resorption and formation. Understanding the mechanisms controlling bone remodeling is fundamental for the understanding of bone failure and to advance treatment of bone disease.

At the nanometric scale, the bone matrix does not appear homogeneous since it is made of interwoven mineralized collagen fibers. In mature human cortical bone, osteons, forming units of bone remodeling, are organized in concentric layered lamellae around canals containing vessel and nerve. The osteons are delimited by a layer of tissue called the cement line. The osteocytes and their processes reside in the lacuno-canalicular system, which is the imprint made by the cells. It is increasingly thought that failure is due to microscopic and ultrastructural properties, therefore the need for quantitative 3D imaging at the nanoscale has arisen [3], [4].

Confocal laser scanning microscopy offers 3D imaging of bone ultrastructure, but the depth of penetration is limited, spatial resolution is anisotropic, it is a scanning technique so data acquisition is slow, and use of advanced staining and sample preparation is necessary [5]. Serial sectioning using a focused ion beam followed by imaging with scanning electron microscopy to image the lacuno-canalicular network has been reported [6], which offers excellent spatial resolution, but is a destructive technique, requiring advanced sample preparation, and relatively long acquisition times.

Different X-ray imaging schemes have been proposed to image the ultrastructure of bone. Transmission X-ray microscopy (TXM) has been used to resolve individual lacunae with canaliculi in mouse cancellous bone [7]. TXM provides high resolution (40 nm) tomographic imaging with quantitative information on the surrounding bone matrix, but the limited field of view (5.5×5.5×5.5 µm^3^) precludes analysis of more than one cell, thus limiting the prospect to perform quantitative studies. Ptychographic imaging with X-rays has recently shown promising results in imaging the lacuno-canalicular network, but limitations are associated with the scanning acquisition which restricts the practical field of view and yields long data acquisition times (reference [8] reports 40 h for 181 projections with 704 scan points each, at a pixel size of 65 nm2 for a field of view of ∼40 µm) The overlapping of the illumination spots also makes ptychography less dose efficient than full-field techniques. Finally the stringent coherence requirements result in inefficient use of the photon flux.

X-ray micro-tomography has proven to be a valuable imaging technique enabling non-destructive 3D analysis of internal structures of materials and is now widely used in biomedical imaging [9]. The high brilliance of synchrotron X-ray sources gives access to sub-micrometric resolution and the possibility to select a monochromatic beam, allowing quantitative imaging of the degree of mineralization, while maintaining short acquisition times. Synchrotron tomography (SR-CT) has proved to be an accurate tool to investigate 3D bone architecture and local mineralization at different hierarchical scales [10]–[12] but has so far not been demonstrated for imaging of the lacuno-canalicular network.

**Figure 1:**
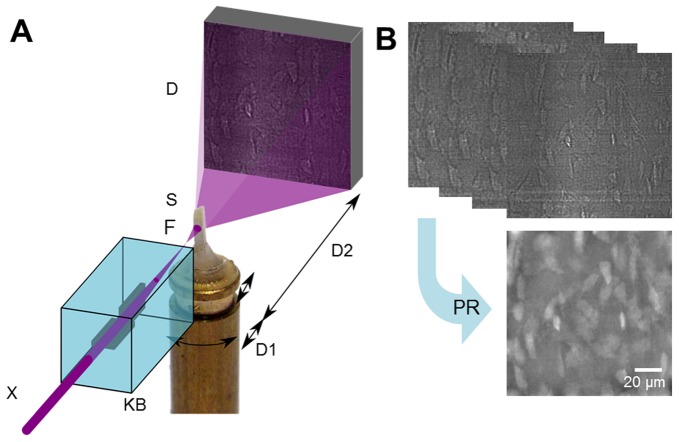
Experimental setup and image reconstruction. [**a**] Schematic of experimental setup. The X-ray beam [X] is monochromatized and focused into a focal spot [F] by X-ray reflective optics [KB]. The sample [S] is positioned on a translation-rotation stage downstream of the focus and imaged onto a stationary detector. Due to the resulting divergent beam, different spot-sample distances [D1] and different free space propagation distances [D2] imply different magnification factors on the detector. [**b**] Images were recorded at four focus-to-sample distances over a complete turn of the sample at 2999 projection angles. The images were used to reconstruct the phase shift at each angle [phase retrieval PR], which was used as input to a tomographic reconstruction algorithm to reconstruct the 3D local mass density.

**Figure 2:**
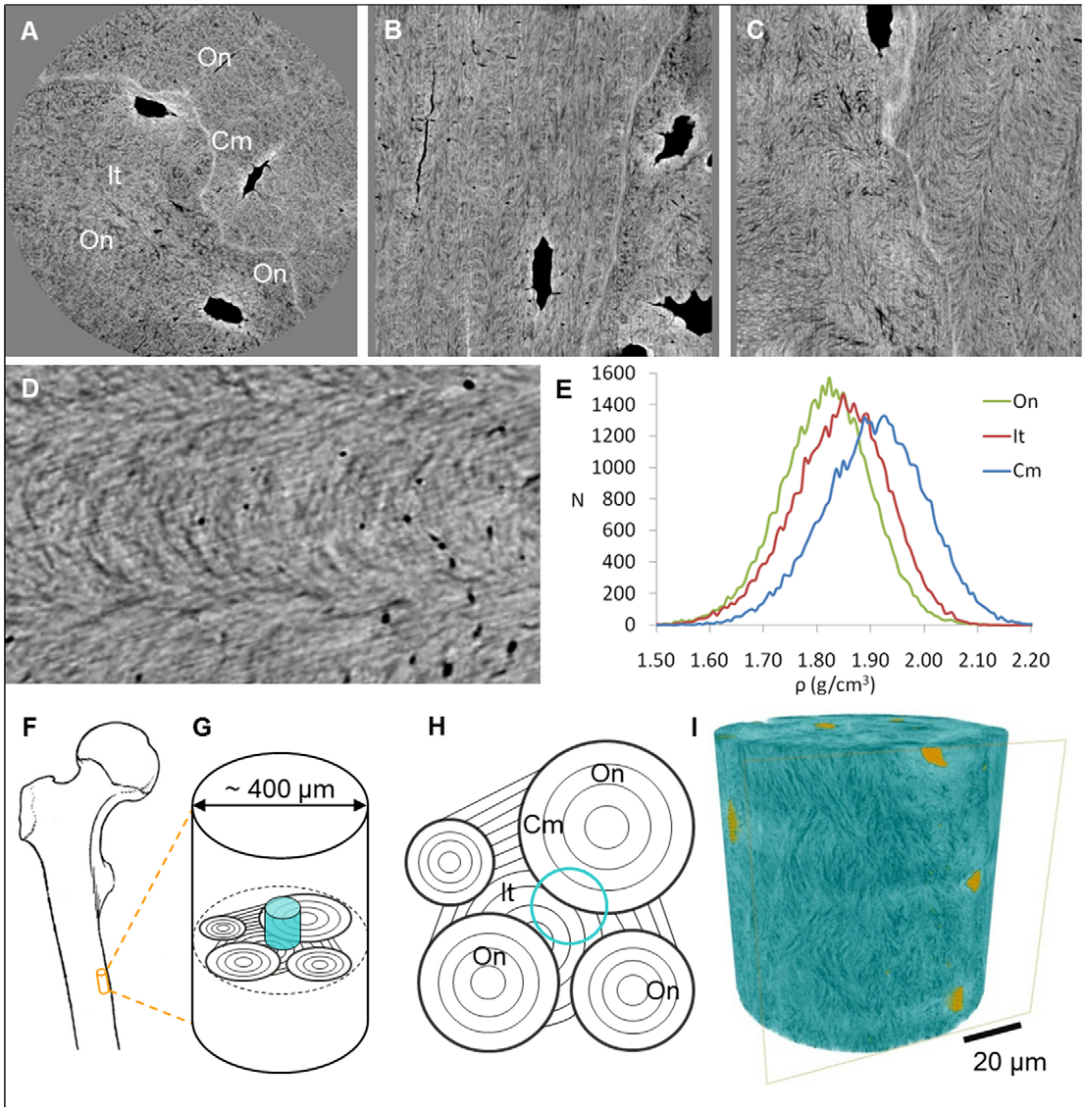
Retrieved information in the reconstructed images. [**a**] Transverse, [**b**] frontal and [**c**] sagittal slices through the images reconstructed from phase data. Grayscale is proportional to local density. Osteocyte lacunae [Lc] and canaliculi [Ca] can clearly be seen. The heterogeneous organization of the matrix by mineralized collagen fibers can also be distinguished [box]. In this sample, a continuous change in collagen orientation can be seen between adjacent lamellae. The cement line [Cm], separating osteonal [On] and interstitial [It] tissue, can clearly be distinguished as more mineralized than the surrounding matrix. Tissue close to osteocyte lacunae is also hypermineralized. [**d**] Zoom on the boxed area in C. Matrix orientation is clearly visible and canaliculi are seen as black dots. [**e**] Mass density histograms in the three tissue types. [**f**] Samples were extracted from the mid diaphysis of a human femur. [**g**] The blue cylinder shows the imaged region inside the sample. [**h**] Schematic of a transverse section showing the organization of lamellar bone in osteons, interstitial tissue and cement lines. Blue circle shows the positioning of A. [**i**] Rendering of the electron density in the sample [blue] and porosity [yellow]. Structures such as osteocyte lacunae [Lc] and canaliculi [Ca], the cement line [Cm] and collagen fibers are revealed.

Here we report the first, to our knowledge, non-destructive true 3D imaging of bone at the ultrastructural level, using X-ray magnified phase tomography. The X-ray beam is monochromatized and focused using X-ray reflective optics to a spot size of 50×50 nm^2^ [13]–[16]. The sample is placed behind the focal spot and imaged onto a scintillator screen and a CCD camera a fixed distance downstream of the focal spot (Fig. 1a). Due to the resulting divergent beam, different magnification factors can be achieved by moving the sample relative to the focal spot. Specimens measuring 0.4×0.4×5 mm^3^ were extracted from the mid-diaphysis of a human femur (Fig. 2f) (Supplementary Methods). The sample is mounted on an air bearing rotation stage to perform tomographic imaging (Fig. 1a).

Due to the large relative propagation distances, phase contrast images are obtained with an exceptional sensitivity for density changes. A series of tomographic scans were recorded at four positions relative to the focal spot. The recorded images are not suitable for direct use for standard tomographic reconstruction, but they allow the calculation of phase shift through phase retrieval. At each projection angle, the four images were used to retrieve the phase using a linear filtering based algorithm [17] (Fig. 1b). Due to the long propagation distance, the non-linear contribution to the contrast is non-negligible. The retrieved phase was further refined using a non-linear conjugate gradient descent algorithm to account for the non-linear contribution in the radiographs and to improve resolution. The retrieved phase maps were input to a tomographic reconstruction algorithm based on filtered backprojection. The resulting tomograms are a reconstruction of the 3D refractive index distribution, being proportional to the local mass density in the object.

## Results

Reconstructed tomographic slices in the transverse (a), frontal (b) and sagittal (c) plane are shown in Fig. 2. These show ultrastructural features in bone in 3D with unprecedented quality. Not only are the osteocyte lacunae (Lc) and canaliculi (Ca) clearly visible, but also features such as the cement line (Cm), the structure of the surrounding matrix and its mineralization. The composite nature of an interwoven mineralized fiber matrix can be clearly seen (Fig. 2a–d, i, Movies S1, S2, S3, S4).

**Figure 3:**
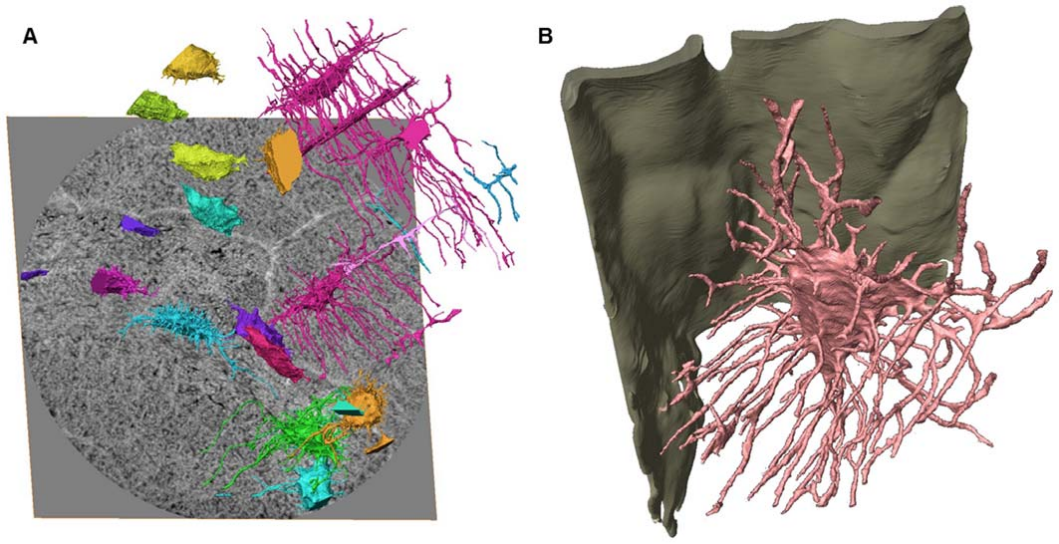
3D renderings of ultrastructural bone features. [**a**] Rendering of osteocyte lacunae and canaliculi in the whole imaged volume overlayed over the bottom slice shown in grayscale. Colors correspond to connected components and grayscale to mass density. Note the difference in structure in the interstitia and osteon: the connected cells are all in the osteonal tissue, the others in the interstitial. The canaliculi are considerably reduced in the interstitia. [**b**] Zoom on the highlighted lacuna in A showing the interaction between the canaliculi [pink] and the cement line [green], and branching of the canaliculi.

In the tomographic images, osteocyte canaliculi enclosing the cellular processes are visible as black spots and striations depending on their orientation relative to the virtual cutting plane (Fig. 2a–d). We remark the flattened lenticular drop shape of the osteocyte lacunae, the top end being wider and rounder and tapering off towards the bottom (Fig. 3). In osteonal tissue the canaliculi are resolved, perfectly maintaining the connectivity of the network, and we can see that most canaliculi branch into two or three a short distance from the lacuna (Fig. 3b). The canalicular network is considerably decreased in the interstitial tissue (Fig. 3a, Movie S5).

The cement line can clearly be seen in the tomograms as a bright line (Fig. 2a–d, i). This is the tissue separating osteons from each other and from interstitial tissue, forming the boundary between the end point of bone resorption and the starting point of new bone formation. From the grayscale images, the local mass density can be directly measured (see Materials and Methods). Histograms of the mass density in three different tissue types measured in subvolumes of 8 µm3 are shown in Fig. 2e. We can see that in this sample, the cement line is hypermineralized; it has a significantly higher density (1.909±0.097 g/cm^3^) than the surrounding osteonal (1.818±0.089 g/cm^3^) and interstitial tissue (1.845±0.090 g/cm^3^) (ANOVA F-test, p<0.01, Post-hoc Tukey's HSD test, p<0.01). The interstitial tissue is also significantly more mineralized than the osteonal tissue (Post-hoc Tukey's HSD test, p<0.01).

Further, in this volume no canaliculi cross the cement line, they rather turn and run parallel with it, in line with what has been previously observed by confocal light scanning microscopy [18]. Here however, we can observe that some canaliculi end at the cement line (Fig. 3b). In the osteonal tissue, lacunae have similar shape and size (286.4±1.2 µm^3^, N = 9) and they are well connected via the canaliculi (canalicular volume over lacunar volume ∼50%). Conversely, in the interstitial tissue all the lacunae are disconnected, with variable shapes and sizes (364.1±63.6 µm^3^), larger in volume than the intra-osteonal lacunae (ratio canalicular volume over lacunar volume ∼6%).

From the tomographic slices (Fig. 2a–c) we can see that, in this sample, the osteonal tissue demonstrates a plywood-like structure. There is a continuous variation of angles of orientation throughout the lamellae, showing the characteristic arc-like structure in slices oblique to the lamellae orientation (Fig. 2c) [19].

## Discussion

We have demonstrated tomographic imaging of bone ultrastructure with 3D X-ray phase nanotomography. This method allows full-field imaging with a large field of view without reverting to scanning techniques. In addition, no special sample preparation is needed. The zoom effect available with the projection setup allows for imaging of a specific region of interest selected inside an extended sample. The method is more sensitive than attenuation based techniques, such as TXM, but also phase based techniques such as ptychography. This method overcomes some of the limitations of the alternative methods for imaging bone tissue at this scale. Still, the proposed method does not reach the highest achievable spatial resolution, it depends critically on the access to a synchrotron facility and relies on a delicate phase retrieval step. However, this technique is very well adapted to 3D studies of the bone nanostructure. This is evidenced by not only revealing the 3D organization of the lacuno-canalicular network, but also the collagen fiber orientation and differences in mineralization between different bone tissue types.

The exact role of the osteocytes in bone remodeling and control of bone mineralization is still unclear. Very little quantitative data on the osteocyte system is available and many interpretations are questionable due to possible artifacts in the techniques used so far [20]. Here, we have shown anatomical features of the LCN, such as precise shape of the lacunae, branching of the canaliculi and the relationship between the LCN and the cement line, unattainable with previously used techniques. The spatial resolution in the image was estimated to be 120–150 nm which seemed appropriate for canaliculi observation. Canaliculi in human bone have been previously measured to be in the range of 0.2–0.9 µm [21]. Further, in Fig. 3, we see either a fully connected network on one side of the cement line and an almost complete absence of canaliculi on the other. If the resolution was insufficient, we would expect to see disconnections and partially vanishing canaliculi. Since the mean space between two canaliculi is much larger than the spatial resolution, and since they produce high contrast they are easily detected.

The cement line is thought to have an important role in limiting damage propagation and the overall stiffness of bone [22]–[24]. It has been disputed whether the cement line is hypomineralized [22], [24] or hypermineralized [25], [26]. Here we can directly measure the density of the different tissues relative to the background, and show that the cement line has a significantly higher mass density than the surrounding osteonal and interstitial tissue.

The ability to directly study the collagen fiber orientation in 3D in the bone opens completely new possibilities for studying bone fragility. Collagen fiber orientation analysis has so far been performed in 2D using scanning electron microscopy [27], [28], transmission electron microscopy [29], atomic force microscopy [30], or indirectly analyzed by polarized light microscopy [31] and Raman spectral mapping [32]. The slices through our 3D images show structures similar to those observed in scanning electron microscopy confirming the identification of these structures [28]. The precise description of the 3D arrangement of collagen fibers as can be obtained with the proposed technique is fundamental for the understanding of the mechanical properties of bone. It should be pointed out that at this very high spatial resolution (60 nm voxel size) the dose delivered to the sample is as high as 8 10^7^ Gy. The specimen does not show any observable modifications, but the high dose currently precludes mechanical testing after X-ray nanotomography [33]. Improvements in the control of the environmental conditions and a reduction of the required dose would be needed to combine X-ray nanotomography and mechanical testing.

Magnified phase tomography allows adequate investigation of the anatomy of the osteon at the collagen fiber level in 3D. Due to this, coupled with the fast acquisition time and simple sample preparation, the proposed methodology is expected to have a major impact in the production of new quantitative data, necessary for advances in bone biology and in designing biomedical and bioinspired materials.

## Materials and Methods

### Sample preparation

Specimens were extracted from the mid-diaphysis of human (women, 87 and 92 years old) femur obtained from a multi-organ collection. A slice of approximately eight millimeters thick was cut perpendicular to the bone axis at the mid-shaft of each femur. Bone samples underwent a treatment (Biobank, Presles en Brie, France) consisting of supercritical carbon dioxide for delipidation, chemical bath in order to eliminate medullary proteins and gamma irradiation for sterilization. After this treatment, samples are considered to be stable in time and can be stored at room temperature. Ethical approval for the samples was granted by the Human Ethics Committee of the “Centre du don des Corps” at the University René Descartes (Paris, France). The tissue donors or their legal guardians provided informed written consent to donate their tissue for investigation, in accord with legal clauses stated in the French Code of Public Health. Small parallelepiped samples (0.4 mm×0.4 mm×5 mm) were then cut with a diamond saw for imaging.

### Imaging set-up

Nano-tomography was performed at the nano-imaging station ID22NI of the ESRF. The technique uses as illumination source the X-ray spot focus produced by dynamically figured multilayer-coated mirrors (Kirkpatrick-Baez crossed mirror geometry). An X-ray energy of 17 keV was selected corresponding to the first harmonic of a single-line undulator (19 mm period U19). No other monochromatization than the one provided by the multilayer coatings was used, assuring a high flux and short acquisition times. The undulator-multilayers system provides a medium monochromaticity of Δ*E*/*E* = 1.6×10^−2^. The sample was set at a small distance downstream of the focus and the transmitted intensity was recorded with a two-dimensional detector set at a large distance from the focus. The geometric magnification *M* of this setup is given by
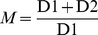
(1)where D1 is the distance between the focal point and the sample, and D2 the distance between the sample and the detector. The radiographs were acquired with a detector system consisting of a high efficiency LSO:Tb luminescent converter screen [34], lens coupled to a large dynamic range and highly efficient Charged-Coupled Device camera (ESRF developed FReLoN camera). A detector area of 1500×1500 pixels and an effective pixel size of 1 µm was used. Radiographs were taken at four sample-source distances (D1 = {32.6, 33.6, 37.6, 47.6} mm) while keeping the detector position fixed at D1+D2 = 525 mm. This geometry results in a final pixel size of 60 nm and a field of view of 90 µm. The sample being significantly larger (0.4 mm sides) than the field of view, local tomography was performed after selecting the relevant region of interest on a low resolution tomography scan (0.32 µm pixel size with a single propagation distance). For tomography, images were recorded at 2999 angular positions of the sample around a vertical rotation axis over one complete turn. The exposure time for each radiograph was 100 ms, resulting in a total acquisition time of 1.9 hours for the tomographic scans at four focus-to-sample distances.

### Image reconstruction

From the data acquired in the four tomographic scans the 3D mass density distribution in the sample is reconstructed. We briefly outline here the different steps of this process. The radiographs recorded in the divergent beam geometry of Fig. 1 are magnified Fresnel diffraction patterns that contain the object information in an entangled form. A phase retrieval step based on radiographs measured at the same angular position but different distances allows to ‘deconvolve’ the image formation process and retrieve the object information. The radiographs depend on the sample through its complex refractive index *n*(*x*,*y*,*z*) and on the settings of the experiment (propagation distances D1, D2 and the X-ray wavelength*λ*). In the present case of hard X-rays the propagation and dynamical effects inside the sample can be neglected, and the wave exiting the sample is modulated in amplitude as
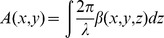
(2)and in phase as
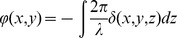
(3)with the absorption index *β* and the refractive index decrement *δ*. These optical properties describe the complex refractive index as
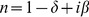
(4)As equations (2) and (3) are projections along the X-ray path, the distributions of *β* and *δ* can be reconstructed with tomography techniques assuming the amplitude and phase modulation can be measured for different angular positions of the sample. The absorption index *β* is proportional to the familiar linear attenuation coefficient *μ* ( = 4π*β*/*λ*). The dominant information resides in the refractive index decrement given by
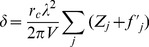
(5)where *r_c_* = 2.8 fm is the classical electron radius. The summation is over all atoms contained in the representative volume *V*; *Z_j_* is their atomic number and *f'_j_* the real part of the wavelength dependent dispersion correction. By selecting an X-ray energy of 17 keV, far above the absorption edges of the elements present in the sample, the dispersion correction is negligible (highest K 1 s binding energy at 4.0 keV for Calcium). Thus the refractive index decrement is simply proportional to the electron density in the sample. As bone tissue is mostly constituted of light elements, the ratio atomic number over atomic mass *Z*/*A* is very close to 1/2 and the refractive index decrement can be well approximated as [35]

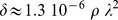
(6)where the mass density *ρ* is expressed in g/cm^3^ and the wavelength *λ* in Å (0.729 at X-ray energy of 17 keV).

For the phase retrieval we use the equivalence between spherical wave illumination and plane wave illumination with a magnification given by equation (1) and the equivalent propagation distance
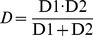
(7)The four radiographs are resampled to a unique magnification corresponding to the plane closest to the focus (pixel size of 60 nm). A first estimate of the phase map is obtained with a linear least squares method weighting correctly the contribution of each of the four propagation distances. An adaptation of the linear method described in [17] is used. Here, we assume proportionality between the phase and amplitude modulation according to a fixed *δ/β* ratio. This supplementary assumption allows to use the absorption information, sensitive to low spatial frequencies, to complement the phase information, mainly sensitive to high spatial frequencies. The high *δ/β* ratio of 202 for cortical bone (ICRU-44) quantifies well the higher sensitivity of phase imaging as compared to attenuation imaging. Due to the large equivalent propagation distances and the high spatial resolution, the non-linear contributions in the image contrast are non-negligible. The retrieved phase was therefore refined using an iterative procedure based on a non-linear conjugate gradient method. This refinement is crucial for the spatial resolution. Ten iteration steps were used. In this part of the phase retrieval, the amplitude and phase modulation were no longer assumed to be proportional, but the amplitude modulation was enforced to contain no high spatial frequency information.

The retrieved phase maps were used as input for tomography reconstruction using a filtered backprojection algorithm. We used the ESRF inhouse developed software PyHST that includes a specific padding scheme adapted to local tomography. According to eq. 6, the reconstruction of the distribution of the refractive index decrement *δ* yields directly the distribution of the mass density *ρ*. To correct for the bias introduced by local tomography, a simple correction of the offset was performed. It is based on the knowledge that the density inside the osteocyte lacunae is equal to zero. The average and standard deviation of the mass density were evaluated in a small region of interest in three different types of tissue. Due to the biological variation within each tissue, the standard deviation is an overestimation of the statistical uncertainty on the experimental data.

### Image analysis

Prior to the segmentation of the lacuno-canalicular network, we used a 3D line enhancement filter [36] to improve the contrast of canaliculi with respect to the bone matrix. This filter is based on eigenvalue decomposition of the second order structure of the image, computed locally. The mutual relations between the eigenvalues permit to identify voxels belonging to a tubular-like structure, with the size given as a parameter. The voxels that are likely to be part of canaliculi are highlighted while the rest of the voxels are darkened. This filter permits to keep the connectivity of the canaliculi while thresholding.

Let 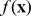 with 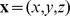, be a 3D image. Because the second order derivatives are sensitive to noise, the image is smoothed with a Gaussian kernel. The second derivatives of the smoothed image, denoted 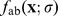, can be calculated as:
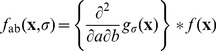
(8)where 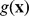 is an isotropic Gaussian function with standard deviation 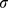
and 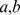 are the row and column indices in the Hessian matrix. The filter may thus be tuned to a specific scale by adjusting 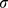.

Let 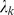 be the eigenvalue with the 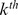
smallest magnitude (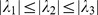) of the Hessian matrix at voxel 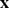. A voxel in an ideal tubular structure is characterized by: 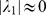; 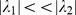; 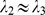. For bright structures, 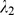 and 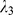
must have negative values. The similarity measure as defined in [36] can be expressed as following:
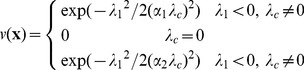
(9)with 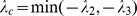.

After filtering and thresholding, we performed an analysis of the connected components [37] in order to quantify the network and to remove residual noise. Unconnected components measuring less than ∼2 µm^3^ were eliminated.

The algorithms for image analysis were implemented in C and C++ languages, based on an in-house library and on ITK library (Kitware). For data visualization and generation of 3D renderings we used the software Avizo (VSG) and ImageJ (NIH).

## Supporting Information

Movie S1 **Movie through the transverse slices in the reconstructed local density distribution.**
(AVI)

Movie S2 **Movie through the frontal slices in the reconstructed local density distribution.**
(AVI)

Movie S3 **Movie through the sagittal slices in the reconstructed local density distribution.**
(AVI)

Movie S4 **Movie showing the 3D rendered local density (blue) and porosity (orange).**
(AVI)

Movie S5 **Movie showing connected components in the porosity (one color for each component) overlaid over a sliding transverse slice through the reconstructed local showing the separation of and differences in osteonal and interstitial tissue.**
(AVI)
